# Variations in the Diagnosis and Management of Benign Paroxysmal Positional Vertigo Among Physician Specialties in Saudi Arabia: Influence of Clinical Experience and Case Exposure

**DOI:** 10.3390/healthcare13151887

**Published:** 2025-08-01

**Authors:** Sarah Alshehri, Abdullah Oudah Al Ahmree, Abdulaziz Qobty, Abdullah Musleh, Khalid A. Alahmari

**Affiliations:** 1 Otolaryngology, Head and Neck Surgery, Department of Surgery, College of Medicine, King Khalid University, Abha 61421, Saudi Arabia; abd.qobty@gmail.com (A.Q.); amusleh@kku.edu.sa (A.M.); 2 Armed Forces Hospital, Abha 62413, Saudi Arabia; abdullah_ala7mary@hotmail.com; 3 Physical Therapy Program, Department of Medical Rehabilitation Sciences, College of Medical Applied Sciences, King Khalid University, Abha 61421, Saudi Arabia; kahmarie@kku.edu.sa

**Keywords:** Benign paroxysmal positional vertigo, diagnostic maneuvers, canalith repositioning, physician specialties, clinical experience

## Abstract

**Background/Objectives**: Benign paroxysmal positional vertigo (BPPV) is the most prevalent vestibular disorder encountered in clinical settings and is highly responsive to evidence-based diagnostic and therapeutic interventions. However, variations in practice patterns among physician specialties can compromise timely diagnosis and effective treatment. Understanding these variations is essential for improving clinical outcomes and standardizing care. This study aimed to assess the diagnostic and treatment practices for BPPV among Ear, Nose, and Throat (ENT) specialists, neurologists, general practitioners, and family physicians in Saudi Arabia and to examine how these practices are influenced by clinical experience and patient case exposure. **Methods**: A cross-sectional, questionnaire-based study was conducted between April 2023 and March 2024 at King Khalid University, Abha, Saudi Arabia. A total of 413 physicians were recruited using purposive sampling. Data were analyzed using IBM SPSS version 24.0. Parametric tests, including one-way ANOVA and chi-square tests, were used to assess differences across groups. A *p*-value of <0.05 was considered statistically significant. **Results**: Overall, all physician groups exhibited limited adherence to guideline-recommended positional diagnostic and therapeutic maneuvers. However, ENT specialists and neurologists demonstrated relatively higher compliance, particularly in performing the Dix–Hallpike test, with 46.97% and 26.79% reporting “always” using the maneuver, respectively (*p* < 0.001, Cramér’s V = 0.22). Neurologists were the most consistent in conducting oculomotor examinations, with 73.68% reporting routine performance (*p* < 0.001, Cramér’s V = 0.35). Epley maneuver usage was highest among neurologists (86.36%) and ENT specialists (77.14%) compared to family physicians (50.60%) and GPs (67.50%) (*p* = 0.044). Physicians with 11–15 years of experience and >50 BPPV case exposures consistently showed a greater use of diagnostic maneuvers, repositioning techniques, and guideline-concordant medication use (betahistine 76.67%; *p* < 0.001). Continuing medical education (CME) participation and the avoidance of unnecessary imaging were also highest in this group (46.67% and 3.33%, respectively; *p* < 0.001). **Conclusions**: Significant inter-specialty differences exist in the management of BPPV in Saudi Arabia. Greater clinical experience and higher case exposure are associated with improved adherence to evidence-based practices. Targeted educational interventions are needed, particularly in primary care, to enhance guideline implementation.

## 1. Introduction

Benign paroxysmal positional vertigo (BPPV) is the most common peripheral vestibular disorder encountered in clinical practice, characterized by brief episodes of vertigo precipitated by changes in head position relative to gravity [1]. It results from dislodged otoconia migrating into one or more semicircular canals, most commonly the posterior canal, leading to abnormal stimulation of the vestibular apparatus [2]. While BPPV is typically benign and treatable, it can substantially impair quality of life, increase the risk of falls, and contribute to significant healthcare utilization if not promptly and accurately managed [3]. Diagnostic confirmation is typically achieved through positional maneuvers such as the Dix–Hallpike test, and effective treatment often involves canalith repositioning procedures (CRPs), notably the Epley maneuver [4]. Evidence-based guidelines consistently advocate for a structured diagnostic and therapeutic approach, emphasizing the avoidance of unnecessary imaging and the limited role of pharmacological interventions, except in cases of persistent symptoms or accompanying vestibular disorders [5]. Despite this, considerable heterogeneity in clinical practice persists across different healthcare settings and physician specialties [6].

The successful management of BPPV relies not only on guideline awareness but also on clinical training, practical exposure, and physician confidence in using vestibular assessment techniques [7]. Prior research has highlighted notable discrepancies in how BPPV is diagnosed and treated among healthcare providers, with some studies reporting suboptimal adherence to recommended maneuvers and a tendency to overuse diagnostic imaging or rely solely on pharmacologic treatment [8]. Differences in physician specialty, access to continuing medical education (CME), and clinical settings have been proposed as contributing factors to these variations [9]. Specialists such as otolaryngologists and neurologists, whose practice scope includes vestibular disorders, are generally more proficient in the use of positional tests and therapeutic maneuvers, whereas general practitioners and family physicians may encounter BPPV less frequently or lack the formal training required to implement evidence-based management [10]. These challenges are further compounded in primary care settings where time constraints and limited access to diagnostic tools may hinder guideline adherence [11]. As a result, patients may be subjected to delayed diagnosis, inappropriate treatment, or unwarranted investigations, thereby compromising clinical outcomes and increasing healthcare costs [12].

Despite the global burden of BPPV and its well-established diagnostic and treatment protocols, there remains a paucity of research evaluating the consistency of clinical practice across specialties, particularly in developing healthcare systems [13]. In the context of Saudi Arabia, where primary care forms the cornerstone of the healthcare delivery system, the extent to which General Practitioners (GPs) and family physicians align with BPPV management guidelines remains underexplored. Furthermore, little is known about how physician demographics, years of clinical experience, and exposure to BPPV cases influence adherence to evidence-based practice in this setting. The existing literature tends to focus on single-specialty populations or hospital-based surveys, limiting the generalizability of findings to broader healthcare systems. The absence of data-driven assessments on diagnostic practices, therapeutic approaches, and perceived barriers within the Saudi healthcare context constitutes a significant gap in the literature. Understanding these variations is critical for identifying professional development needs and implementing targeted educational strategies to improve the quality of vestibular care across all levels of clinical practice.

The present study was designed to address this knowledge gap by evaluating the diagnostic and treatment practices for BPPV among Ear, Nose, and Throat (ENT) specialists, neurologists, GPs, and family physicians in Saudi Arabia. The study also aimed to examine how physician demographics, clinical experience, and BPPV case exposure relate to adherence to evidence-based guidelines.

## 2. Materials and Methods

### 2.1. Design and Setting

**Figure 1:**
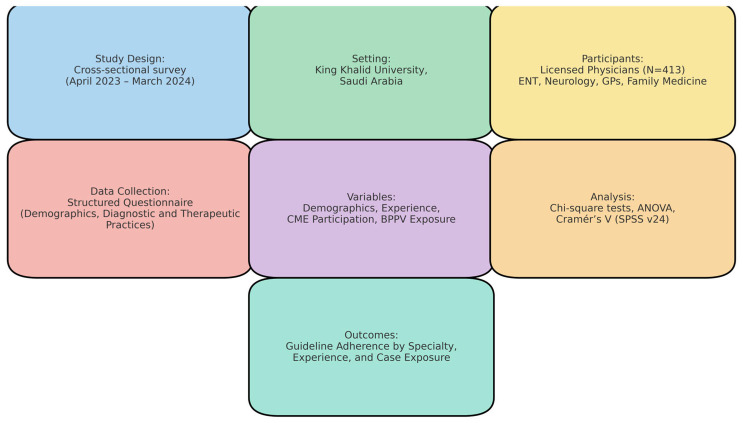
Schematic representation of the study design, participant characteristics, data collection domains, and key analytical outcomes.

This cross-sectional study was conducted between April 2023 and March 2024 at the Department of Physical Therapy, King Khalid University, Abha, Saudi Arabia. A schematic overview of the study design, including the sampling structure, variables analyzed, and outcome domains, is presented in Figure 1 to visually support the methodological flow. The research protocol was reviewed and approved by the institutional ethical committee (KKU, DSR, ECM#2025-713), ensuring full compliance with ethical standards. All participating physicians were provided with comprehensive information about the study’s purpose and procedures, and written informed consent was obtained from each respondent prior to participation. The study strictly adhered to the ethical principles outlined in the Declaration of Helsinki, including voluntary participation, confidentiality of data, and the right to withdraw at any stage without consequence.

### 2.2. Participants

Participants in this study included licensed physicians from four clinical specialties—ENT, neurology, general practice, and family medicine—actively practicing in various healthcare settings across Saudi Arabia. Eligible participants were recruited from both governmental and private healthcare institutions, including regional hospitals, primary care centers, and tertiary-level clinics. The recruitment process was conducted through professional networks, institutional collaborations, and targeted invitations distributed via email and in-person contact during departmental meetings. The selection aimed to ensure a representative distribution across specialties, years of experience, and work environments. The study employed purposive sampling to target physicians likely to encounter patients with BPPV, as defined by established clinical diagnostic criteria.

The diagnostic framework used to guide participant eligibility was based on the clinical presentation of BPPV as outlined by international consensus guidelines: patients typically experience brief episodes of vertigo precipitated by changes in head position, confirmed by positive responses to positional tests such as the Dix–Hallpike or roll test. While direct diagnosis of BPPV was not required from participants, their clinical familiarity with its diagnostic and therapeutic procedures formed a prerequisite for inclusion. Thus, participants were included if they had managed at least one suspected or confirmed case of BPPV within the preceding year and were involved in patient care relevant to vertigo or dizziness symptoms.

Inclusion criteria comprised licensed physicians from the included specialties with at least one year of post-graduate experience and prior exposure to at least one vertigo patient in the past year. Exclusion criteria included medical interns, residents in training not yet engaged in independent clinical decision-making, physicians who had never managed a case of vertigo, and those unwilling to provide informed consent. Respondents who submitted incomplete questionnaires or failed to meet the minimum case exposure threshold were also excluded from the final analysis. Incomplete questionnaires and those with inconsistent responses were excluded from the analysis, resulting in a final sample of 413 valid entries.

The selection of participants was further refined to capture variability in clinical practice based on years of experience, and the volume of BPPV cases managed. This stratification enabled comparative analysis of diagnostic and treatment patterns across different levels of clinical exposure and expertise. Through this rigorous recruitment and eligibility process, the study ensured that only qualified and contextually relevant physicians were included, thereby enhancing the reliability and applicability of the findings.

### 2.3. Variables

This study investigated several key variables related to the diagnosis and management of BPPV among physicians, categorized into three main domains: demographic and professional characteristics, diagnostic and therapeutic practices, and factors influencing adherence to clinical guidelines.

#### 2.3.1. Demographic and Professional Variables

These included age, gender, specialty (ENT, neurology, general practice, or family medicine), years of clinical experience, and work setting (regional hospital, private clinic, university hospital, or other healthcare facility). These variables were self-reported using a structured questionnaire. Years of experience were further stratified into four categories: <5 years, 6–10 years, 11–15 years, and ≥16 years. Similarly, the number of BPPV cases managed in the past year were used to estimate clinical exposure and were categorized into <10 cases, 10–50 cases, and >50 cases.

#### 2.3.2. Diagnostic Practices

Diagnostic practice variables included the frequency of positional maneuvers, categorized as ‘Always,’ ‘Rarely,’ or ‘Never,’ with definitions provided in the questionnaire. The reliability of these self-reports was enhanced by providing brief, standardized definitions of each maneuver in the questionnaire. These responses served as categorical outcome variables reflecting guideline adherence in BPPV diagnosis.

#### 2.3.3. Therapeutic Practices

Treatment-related variables were assessed through questions on the use of CRPs, including the Epley, Semont, and Barbecue maneuvers [14]. Participants indicated which maneuvers they regularly applied in clinical settings. Medication use was assessed through selection options for betahistine, cinnarizine, gencine, or none. Additionally, participants were asked whether they recommended home-based exercises, specifying Brandt–Daroff exercises, informal sources such as YouTube, or no exercises at all. These therapeutic responses were categorized and analyzed based on specialty and experience level to identify patterns of evidence-based management.

#### 2.3.4. Guideline Adherence and Influencing Factors

To assess adherence to clinical guidelines, a composite measure was derived based on three primary practices: performance of diagnostic maneuvers, use of CRPs, and avoidance of unnecessary imaging. Respondents also reported whether they had participated in CME activities related to vestibular disorders in the previous 12 months. These variables were dichotomized (Yes/No) and used to evaluate the relationship between physician exposure and training with their compliance with standardized management protocols. Additionally, reported barriers to proper diagnostic evaluation—specifically, lack of time, lack of knowledge, and fear of provoking vertigo—were included as categorical variables.

All data were collected through a structured, self-administered electronic questionnaire developed by the study investigators. The questionnaire was reviewed by a panel of experts in neurology, otolaryngology, and clinical research to ensure content validity. The questionnaire was translated into Arabic using forward-backward translation by bilingual experts to ensure semantic equivalence. The pilot was conducted with 10 physicians across the four specialties to assess item clarity and response validity. Feedback led to minor linguistic refinements but no structural changes. Data were entered and managed in a secure electronic database, with each variable coded consistently for statistical analysis. The detailed and standardized measurement of variables enables replication of this study across similar healthcare settings and supports meaningful comparisons in future research.

### 2.4. Sample Size Calculation

The sample size for this study was calculated prior to data collection using G*Power statistical software (version 3.1.9.7, Heinrich Heine University, Düsseldorf, Germany) to ensure adequate power for detecting statistically significant differences across physician specialties in their diagnostic and therapeutic practices for BPPV. Based on an assumed medium effect size (Cramér’s V = 0.25), the required minimum sample size to detect statistically significant differences among groups using a chi-square test was calculated to be 260 participants, with an alpha level of 0.05 and statistical power of 0.90. To ensure robustness against potential non-response or incomplete data, this figure was increased by 20%, yielding a final target sample size of 312 participants.

### 2.5. Data Analysis

All statistical analyses were performed using IBM SPSS Statistics version 24.0 (IBM Corp., Armonk, NY, USA). Descriptive statistics were used to summarize the demographic and professional characteristics of participants across specialties, including means and standard deviations for continuous variables and frequencies and percentages for categorical variables. As the data met assumptions of normality, parametric tests were employed. One-way analysis of variance (ANOVA) was used to compare means across more than two groups for continuous variables such as years of experience and number of BPPV cases managed. Chi-square tests were applied to assess associations between physician specialty and categorical outcomes such as the use of diagnostic maneuvers, therapeutic techniques, medication prescription, and CME participation. Where significant associations were found, post hoc comparisons with Bonferroni correction were conducted to identify specific group differences. A *p*-value of <0.05 was considered statistically significant for all analyses. This analytical approach allowed for the precise evaluation of the study objectives, including comparisons of clinical practices by specialty, experience, and BPPV exposure.

## 3. Results

Table 1 presents significant demographic and professional variations among ENT specialists, neurologists, general practitioners, and family physicians practicing in Saudi Arabia. Gender distribution varied notably, with neurologists showing the highest proportion of female respondents, while ENT specialists were predominantly male. Age distribution differed significantly, with neurologists skewing younger (majority in the 30–39 age group) compared to family physicians, who had a higher representation in the 40–49 age range. Years of experience also varied markedly, with ENT specialists and neurologists largely comprising early-career professionals (<5 years), whereas family physicians reported longer clinical experience, especially in the 11–15-year range. Work settings differed across specialties; most ENT specialists and neurologists operated in non-regional or undefined settings, while the majority of GPs and family physicians were based in regional healthcare facilities. Notably, ENT specialists had significantly higher participation in CME activities compared to other groups, among whom CME engagement was exceedingly low.

Table 2 highlights substantial variability in the diagnostic approaches to BPPV among physician specialties, with ENT specialists (46.97%) and neurologists (26.79%) generally demonstrating more adherence to recommended practices than general practitioners and family physicians. ENT specialists showed a higher frequency in performing diagnostic maneuvers such as the Dix–Hallpike, head thrust, and head shake tests, while neurologists showed a notably higher frequency of oculomotor examinations. In contrast, GPs and family physicians more often reported rarely or never performing these assessments, particularly balance and gait evaluations, which were infrequently used outside of the neurology and ENT groups. The strongest disparities were observed in the use of balance and gait assessments, supported by high Cramér’s V values, indicating meaningful differences across specialties. Reported barriers further explained these trends: ENT specialists and neurologists cited lack of time as the predominant limitation, whereas GPs and family physicians more commonly pointed to lack of knowledge as the main impediment to implementing proper diagnostic procedures.

Table 3 demonstrates considerable inter-specialty variation in the therapeutic management of BPPV, particularly in the use of repositioning maneuvers, medication prescriptions, and home exercise recommendations. Neurologists and ENT specialists reported the highest rates of Epley maneuver utilization, while family physicians had the lowest usage and were most likely to report not performing any canalith repositioning procedure. Betahistine was the most commonly prescribed medication across all groups, especially among neurologists and GPs, whereas family physicians more frequently prescribed no medication or opted for Gencine. Recommendations for home exercises also varied, with ENT specialists more frequently advocating for Brandt–Daroff exercises, in contrast to the minimal use observed among neurologists. Family physicians and GPs more often recommended no home exercise, and reliance on informal resources such as YouTube was minimal across all specialties. The data suggest a trend of more guideline-concordant treatment practices among ENT specialists and neurologists, with GPs and family physicians showing less consistent application of recommended therapeutic modalities.

Table 4 reveals that both clinical experience and exposure to BPPV cases significantly influence diagnostic and treatment practices among physicians. Physicians with greater experience (11–15 years) and higher case exposure (>50 patients) were more likely to consistently perform diagnostic maneuvers and utilize repositioning techniques, particularly the Epley maneuver. In contrast, those with less experience or lower exposure reported more infrequent use of diagnostic assessments and a higher reliance on passive management, including prescribing no treatment. Medication use patterns also varied, with more experienced and higher-exposure groups favoring targeted pharmacological interventions like betahistine and cinnarizine, while less experienced physicians more often reported prescribing no medication. Notably, Cramér’s V values indicate moderate associations, particularly for medication practices, suggesting that familiarity with BPPV cases may promote more guideline-concordant management.

**Figure 2:**
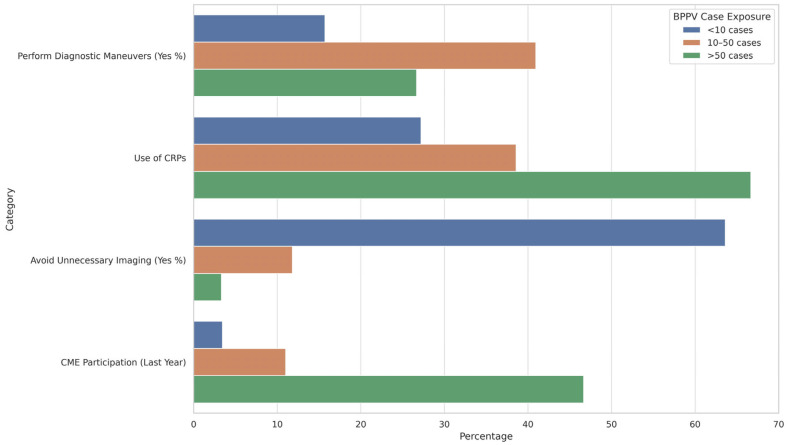
Guideline Adherence and Clinical Practice Based on BPPV Case Exposure.

Figure 2 indicates that higher exposure to BPPV cases is positively associated with improved adherence to clinical guidelines. Physicians managing more than 50 cases of BPPV were significantly more likely to utilize canalith repositioning procedures (CRPs) and participate in CME. This group also showed reduced reliance on unnecessary imaging, aligning with evidence-based practices. In contrast, those with lower case exposure (<10 cases) demonstrated limited use of CRPs, minimal participation in CME, and a markedly higher rate of unnecessary imaging, highlighting a gap in guideline-concordant care among less experienced or less exposed clinicians.

**Figure 3:**
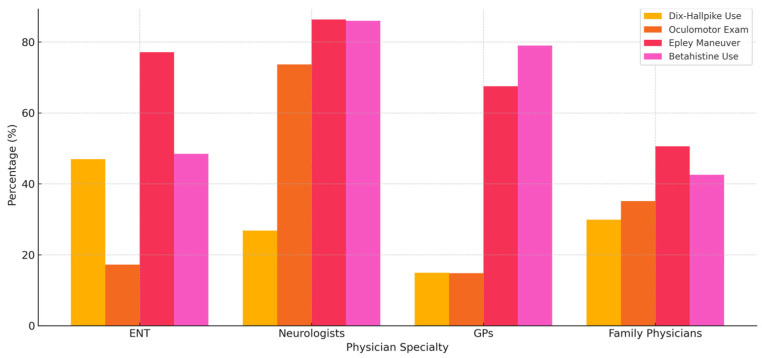
Comparison of diagnostic and treatment practices by physician specialty, including frequency of Dix–Hallpike use, oculomotor examination, Epley maneuver application, and betahistine prescription.

Additionally, Figure 3 provides a comparative visualization of diagnostic and treatment adherence by physician specialty. This summary chart highlights the proportion of physicians consistently performing key maneuvers or prescribing recommended medications, offering a concise view of inter-specialty differences and reinforcing the need for specialty-specific educational interventions.

## 4. Discussion

The present study aimed to evaluate the diagnostic and therapeutic practices employed for BPPV across diverse physician specialties in Saudi Arabia, with a focus on adherence to evidence-based guidelines and the influence of clinical experience and case exposure. The findings revealed significant variability in clinical practices, with fewer than 50% of participants across all groups performing the recommended diagnostic maneuvers and therapeutic interventions. However, ENT specialists and neurologists demonstrated higher adherence compared to general practitioners and family physicians. Diagnostic practices such as the Dix–Hallpike test and oculomotor evaluations were more consistently performed by specialists, while generalists frequently reported barriers such as lack of knowledge. It is important to recognize that these maneuvers are typically interpreted in conjunction with a comprehensive neurotologic examination and detailed patient history. Variations in training and expertise likely influence not only the frequency of use but also the diagnostic confidence and interpretation of these clinical findings. Furthermore, limited access to diagnostic tools in primary care settings may compound these disparities, contributing to the differences observed in guideline adherence. Furthermore, increased clinical experience (particularly in the 11–15-year range) and greater exposure to BPPV cases (>50 patients) were positively associated with the more consistent use of repositioning techniques, reduced reliance on unnecessary imaging, and higher participation in CME, underscoring the role of experiential learning in promoting guideline-concordant care.

The observed variations in diagnostic practices for BPPV among physician specialties can be largely explained by differences in demographic and professional characteristics. ENT specialists and neurologists, who comprised a younger and predominantly early-career cohort, were more likely to engage in evidence-based diagnostic maneuvers such as the Dix–Hallpike, head thrust, and oculomotor examinations. Their greater representation in non-regional or tertiary healthcare settings may facilitate access to specialized equipment and training, as well as institutional support for guideline-concordant practice. Additionally, the significantly higher rates of CME participation among ENT specialists suggest a stronger institutional culture of lifelong learning, which likely contributes to their higher diagnostic proficiency [15]. In contrast, general practitioners and family physicians, who were more experienced and primarily based in regional settings, reported limited use of diagnostic maneuvers and cited lack of knowledge as a primary barrier [7]. This highlights a potential gap in ongoing professional development and exposure to specialized vestibular disorders in primary care environments.

These findings align with the prior literature that underscores the influence of specialty training and education on BPPV management. While the observed trends may be expected based on specialty roles, this study provides quantitative confirmation of their extent within a national healthcare system, revealing specific deficiencies in generalist practice. Such data are essential for shaping targeted CME efforts, resource allocation, and clinical governance strategies. Nevertheless, future qualitative work is needed to explore the contextual and perceptual factors that influence these patterns and enrich the interpretability of survey-based results. Lloyd et al. [16] reported that clinicians in neurology and otolaryngology were significantly more likely to adhere to guideline-recommended positional testing and therapeutic maneuvers compared to generalists. Similarly, Ledger et al. [17] identified inadequate training and infrequent CME as major obstacles to the accurate diagnosis of BPPV among primary care providers. Christy et al. [18] also emphasized the importance of early recognition and appropriate testing, noting that delayed diagnosis often occurred in settings with limited vestibular expertise. These studies corroborate the present results, suggesting that both structural and educational factors—such as healthcare setting, clinical focus, and access to CME—play a critical role in shaping the quality of BPPV care across specialties [7].

The results of this study underscore the significant role of both clinical experience and BPPV case exposure in shaping diagnostic and therapeutic decision-making among physicians. Those with greater experience (particularly in the 11–15-year range) and higher case exposure (>50 patients) demonstrated more frequent and consistent use of recommended diagnostic maneuvers and therapeutic interventions, notably the Epley maneuver. These groups also favored targeted pharmacologic agents such as betahistine and cinnarizine, in contrast to their less experienced counterparts, who more frequently prescribed no medication or resorted to less recommended practices [19]. This pattern extended to adherence behaviors, where greater exposure was linked to increased CME participation and reduced reliance on unnecessary imaging, suggesting a stronger alignment with clinical guidelines [20]. These findings highlight the importance of both experiential learning and continuing education in fostering evidence-based BPPV management.

These observations are supported by previous research emphasizing the positive correlation between clinical exposure and guideline adherence in vestibular disorders [21]. Özgirgin et al. [3] reported that familiarity with BPPV significantly influenced the use of canalith repositioning procedures and minimized reliance on diagnostic imaging. Similarly, Stephan et al. [22] identified a lack of case exposure as a principal barrier to timely and appropriate BPPV management in primary care. Kerber et al. [23] also found that physicians with higher clinical exposure were more likely to engage in CME and apply evidence-based practices. These studies align with the current findings, reinforcing the conclusion that direct patient experience and structured educational engagement are critical in enhancing the quality of care delivered for BPPV [23,24].

### 4.1. Clinical Significance

The clinical significance of this study lies in its detailed examination of the disparities in BPPV management practices among physician specialties and levels of experience within the Saudi Arabian healthcare context [25]. Notably, the findings revealed that adherence to evidence-based diagnostic and therapeutic protocols—such as the use of positional maneuvers, canalith repositioning techniques, and appropriate pharmacologic treatments—was generally suboptimal across all groups. However, ENT specialists and neurologists showed relatively greater compliance compared to general practitioners and family physicians, highlighting the need for targeted educational interventions and standardized clinical guidelines to improve BPPV management outcomes [25]. Furthermore, the findings emphasize that increased clinical experience and greater exposure to BPPV cases are positively associated with improved diagnostic accuracy, reduced reliance on unnecessary imaging, and higher participation in CME. These insights underline the pressing need for targeted educational interventions and structured CME programs, particularly in primary care settings, to bridge existing knowledge gaps and ensure standardized, guideline-concordant care for patients with BPPV across all levels of the healthcare system. We recommend incorporating structured vestibular assessment training into family medicine and general practice residency curricula, establishing mandatory CME credits on BPPV as part of license renewal requirements, and developing national clinical protocols endorsed by medical boards or specialty societies. Digital CME platforms and simulation-based workshops may also improve accessibility and engagement for physicians in remote or resource-limited settings.

### 4.2. Limitations

This study is limited by its reliance on self-reported data, which may introduce response bias and potentially overestimate adherence to BPPV guidelines due to recall or social desirability effects. The absence of objective measures—such as chart reviews, direct observation, or simulated assessments—limits the accuracy of the reported practices. Additionally, the cross-sectional design restricts the ability to infer causal relationships between physician characteristics and guideline adherence. The sample’s overrepresentation of early-career physicians, particularly in ENT and neurology, may also limit generalizability to more experienced cohorts. Institutional factors such as access to diagnostic tools, support for CME participation, and clinic-level infrastructure were not assessed but may significantly influence clinical behavior. These variables likely interact with physician experience and specialty to shape practice patterns and should be explored in future studies. Longitudinal designs evaluating behavior change following educational interventions could provide insights into causality and sustainability. Furthermore, incorporating qualitative interviews with primary care physicians may uncover contextual barriers—such as time constraints, resource limitations, or perceived diagnostic complexity—that are not easily captured through quantitative methods.

## 5. Conclusions

This study provides a comprehensive evaluation of BPPV management practices among physicians in Saudi Arabia, revealing substantial variability across specialties and levels of clinical experience. Notably, the findings revealed that adherence to evidence-based diagnostic and therapeutic protocols—such as the use of positional maneuvers, canalith repositioning techniques, and appropriate pharmacologic treatments—was generally suboptimal across all groups. However, ENT specialists and neurologists showed relatively greater compliance compared to general practitioners and family physicians, highlighting the need for targeted educational interventions and the development of standardized clinical guidelines to improve BPPV management outcomes. Higher clinical experience and BPPV case exposure were strongly associated with a more consistent use of appropriate diagnostic and treatment strategies, greater CME participation, and the reduced use of unnecessary imaging. These findings underscore the importance of targeted educational efforts to enhance BPPV management, particularly in primary care settings. Future studies should use objective methods—such as clinical audits, chart reviews, or direct observation—to validate self-reported practices and minimize bias. Interventional research assessing the impact of CME or standardized care pathways, alongside qualitative or mixed-methods approaches exploring reasons for non-adherence, would provide deeper insight into barriers and support the design of targeted educational and system-level interventions.

## Figures and Tables

**Table 1:** Demographic and Professional Characteristics of Respondents.

Characteristic	ENT (n = 64)	Neurologists (n = 57)	GPs (n = 112)	Family Physicians (n = 180)	*p*-Value
Gender (M/F)					
Female	26 (39.39%)	37 (64.91%)	55 (48.25%)	89 (49.17%)	0.042
Male	40 (60.61%)	20 (35.09%)	59 (51.75%)	92 (50.83%)	0.034
Age Group (% in each)					
<30	23 (34.85%)	5 (8.77%)	24 (21.05%)	61 (33.70%)	<0.001
<30–39	36 (54.55%)	45 (78.95%)	58 (50.88%)	67 (37.02%)	<0.001
40–49	5 (7.58%)	4 (7.02%)	7 (6.14%)	50 (27.62%)	<0.001
50+	2 (3.03%)	3 (5.26%)	25 (21.93%)	3 (1.66%)	<0.001
Years of Experience					
<5	42 (63.64%)	24 (42.11%)	32 (28.07%)	59 (32.60%)	<0.001
6–10	17 (25.76%)	28 (49.12%)	72 (63.16%)	42 (23.20%)	<0.001
11–15	3 (4.55%)	4 (7.02%)	8 (7.02%)	69 (38.12%)	<0.001
≥16	4 (6.06%)	1 (1.75%)	2 (1.75%)	11 (6.08%)	<0.001
Work Setting					
Other	53 (80.30%)	49 (85.96%)	22 (19.30%)	31 (17.51%)	<0.001
Private	5 (7.58%)	2 (3.51%)	12 (10.53%)	36 (20.34%)	<0.001
Regional	6 (9.09%)	6 (10.53%)	80 (70.18%)	102 (57.63%)	<0.001
Univ. Hosp	2 (3.03%)	0 (0.00%)	0 (0.00%)	8 (4.52%)	<0.001
CME Participation (Yes %)					
No	41 (62.12%)	56 (98.25%)	109 (95.61%)	175 (96.69%)	<0.001
Yes	25 (37.88%)	1 (1.75%)	5 (4.39%)	6 (3.31%)	<0.001

ENT: Ear, Nose, and Throat; GP: General Practitioner; CME: Continuing Medical Education; Univ. Hosp: University Hospital.

**Table 2:** Diagnostic Practices and Reported Barriers Across Physician Specialties.

	Variable	ENT (%)	Neurologists (%)	GPs (%)	Family Physicians (%)	*p*-Value	Cramér’s V
Perform Diagnostic Maneuvers	Always	31 (46.97%)	15 (26.79%)	17 (14.91%)	38 (29.92%)	<0.001	0.22
	Rarely	31 (46.97%)	31 (55.36%)	56 (49.12%)	76 (59.84%)	<0.001	0.25
	Never	4 (6.06%)	10 (17.86%)	41 (35.96%)	13 (10.24%)	<0.001	0.24
Perform Oculomotor Exam	Always	10 (17.24%)	42 (73.68%)	16 (14.81%)	51 (35.17%)	<0.001	0.35
	Rarely	21 (36.21%)	14 (24.56%)	15 (13.89%)	27 (18.62%)	<0.001	0.36
	Never	27 (46.55%)	1 (1.75%)	77 (71.30%)	67 (46.21%)	<0.001	0.38
Head Thrust Test	Always	23 (35.38%)	8 (14.55%)	4 (4.04%)	11 (6.63%)	<0.001	0.33
	Rarely	35 (53.85%)	12 (21.82%)	15 (15.15%)	51 (30.72%)	<0.001	0.32
	Never	7 (10.77%)	35 (63.64%)	80 (80.81%)	104 (62.65%)	<0.001	0.35
Head Shake Test	Always	28 (43.08%)	10 (17.54%)	8 (9.09%)	17 (18.09%)	<0.001	0.36
	Rarely	29 (44.62%)	8 (14.04%)	38 (43.18%)	55 (58.51%)	<0.001	0.34
	Never	8 (12.31%)	39 (68.42%)	42 (47.73%)	22 (23.40%)	<0.001	0.32
Balance Examination	Always	21 (31.82%)	27 (48.21%)	3 (2.94%)	10 (6.99%)	<0.001	0.45
	Rarely	26 (39.39%)	27 (48.21%)	7 (6.86%)	39 (27.27%)	<0.001	0.41
	Never	19 (28.79%)	2 (3.57%)	92 (90.20%)	94 (65.73%)	<0.001	0.43
Gait Assessment	Always	6 (11.11%)	28 (49.12%)	7 (11.67%)	24 (26.37%)	<0.001	0.32
	Rarely	27 (50.00%)	28 (49.12%)	18 (30.00%)	45 (49.45%)	<0.001	0.34
	Never	21 (38.89%)	1 (1.75%)	35 (58.33%)	22 (24.18%)	<0.001	0.33
Main Barriers	Lack of time	54 (81.82%)	48 (84.21%)	6 (5.31%)	8 (4.57%)	<0.001	0.48
	Lack of knowledge	4 (6.06%)	5 (8.77%)	70 (61.95%)	80 (45.71%)	<0.001	0.42
	Fear of provoking vertigo	4 (6.06%)	2 (3.51%)	9 (7.96%)	15 (8.57%)	<0.001	0.41

ENT: Ear, Nose, and Throat; GP: General Practitioner. Note: Cramér’s V is a statistical measure of the strength of association between two categorical variables. It is based on the Chi-square test statistic. It adjusts for the size of the contingency table. It ranges from 0 (no association) to 1 (perfect association).

**Table 3:** Treatment Practices for BPPV by Physician Specialty.

	Treatment Variable	ENT (%)	Neurologists (%)	GPs (%)	Family Physicians (%)	*p*-Value	Cramér’s V
Use of Epley/Semont/Barbecue Maneuvers	Epley	27 (77.14%)	19 (86.36%)	27 (67.50%)	42 (50.60%)	0.044	0.14
	Semont	1 (2.86%)	1 (4.55%)	2 (5.00%)	11 (13.25%)	0.044	0.14
	Barbecue	2 (5.71%)	1 (4.55%)	3 (7.50%)	4 (4.82%)	0.044	0.16
	None	5 (14.29%)	1 (4.55%)	8 (20.00%)	26 (31.33%)	0.044	0.18
Prescribe Betahistine/Cinnarizine/Gencine/None	Betahistine	32 (48.48%)	49 (85.96%)	90 (78.95%)	77 (42.54%)	<0.001	0.22
	Cinnarizine	26 (39.39%)	5 (8.77%)	10 (8.77%)	22 (12.15%)	<0.001	0.23
	Gencine	4 (6.06%)	3 (5.26%)	7 (6.14%)	39 (21.55%)	<0.001	0.25
	None	4 (6.06%)	0 (0.00%)	7 (6.14%)	43 (23.76%)	<0.001	0.27
Recommend Home Exercise (Yes/Rarely/Never)	Yes	19 (47.50%)	2 (16.67%)	9 (21.95%)	26 (27.96%)	0.008	0.21
	Rarely	8 (20.00%)	5 (41.67%)	24 (58.54%)	48 (51.61%)	0.008	0.20
	Never	13 (32.50%)	5 (41.67%)	8 (19.51%)	19 (20.43%)	0.008	0.23
Type (Brandt–Daroff/YouTube/None)	Brandt–Daroff	26 (39.39%)	1 (1.75%)	15 (13.16%)	53 (29.28%)	<0.001	0.22
	YouTube	10 (15.15%)	5 (8.77%)	16 (14.04%)	22 (12.15%)	<0.001	0.24
	None	30 (45.45%)	51 (89.47%)	83 (72.81%)	106 (58.56%)	<0.001	0.23

ENT: Ear, Nose, and Throat; GP: General Practitioner. Note: Cramér’s V is a statistical measure of the strength of association between two categorical variables. It is based on the Chi-square test statistic.

**Table 4:** Impact of Clinical Experience and BPPV Exposure on Management Practices.

	Practice Variable	<5 years/<10 pts (%)	6–10 years/10–50 pts (%)	11–15 years/>50 pts (%)	≥16 years (%)	*p*-Value	Cramér’s V
Perform Diagnostic Maneuvers (Experience)	Always	7 (14.00%)	35 (22.58%)	57 (38.00%)	2 (25.00%)	<0.001	0.13
	Never	4 (8.00%)	40 (25.81%)	22 (14.67%)	2 (25.00%)	<0.001	0.15
	Rarely	39 (78.00%)	80 (51.61%)	71 (47.33%)	4 (50.00%)	<0.001	0.14
Use of CRPs (Experience)	Barbecue	0 (0.00%)	3 (5.08%)	7 (6.67%)	0 (0.00%)	0.866	0.06
	Epley	8 (57.14%)	36 (61.02%)	70 (66.67%)	1 (50.00%)	0.866	0.06
	None	5 (35.71%)	15 (25.42%)	19 (18.10%)	1 (50.00%)	0.866	0.07
	Semont	1 (7.14%)	5 (8.47%)	9 (8.57%)	0 (0.00%)	0.866	0.09
Prescribe Medication (Experience)	Betahistine	57 (67.86%)	110 (69.18%)	75 (47.77%)	6 (33.33%)	<0.001	0.21
	Cinnarizine	6 (7.14%)	21 (13.21%)	34 (21.66%)	2 (11.11%)	<0.001	0.24
	Gencine	3 (3.57%)	18 (11.32%)	32 (20.38%)	0 (0.00%)	<0.001	0.23
	None	18 (21.43%)	10 (6.29%)	16 (10.19%)	10 (55.56%)	<0.001	0.26
Perform Diagnostic Maneuvers (Exposure)	Always	52 (41.60%)	41 (19.62%)	8 (27.59%)		<0.001	0.22
	Never	14 (11.20%)	53 (25.36%)	1 (3.45%)		<0.001	0.23
	Rarely	59 (47.20%)	115 (55.02%)	20 (68.97%)		<0.001	0.24
Use of CRPs (Exposure)	Barbecue	3 (5.00%)	7 (7.07%)	0 (0.00%)		0.041	0.17
	Epley	43 (71.67%)	53 (53.54%)	19 (90.48%)		0.041	0.19
	None	11 (18.33%)	28 (28.28%)	1 (4.76%)		0.041	0.16
	Semont	3 (5.00%)	11 (11.11%)	1 (4.76%)		0.041	0.18
Prescribe Medication (Exposure)	Betahistine	68 (53.54%)	157 (60.15%)	23 (76.67%)		<0.001	0.22
	Cinnarizine	37 (29.13%)	20 (7.66%)	6 (20.00%)		<0.001	0.21
	Gencine	15 (11.81%)	38 (14.56%)	0 (0.00%)		<0.001	0.25
	None	7 (5.51%)	46 (17.62%)	1 (3.33%)		<0.001	0.23

BPPV: Benign Paroxysmal Positional Vertigo; CRP: Canalith Repositioning Procedure. Note: Cramér’s V is a statistical measure of the strength of association between two categorical variables. It is based on the Chi-square test statistic.

## Data Availability

The data presented in this study are openly available in Zenodo at https://doi.org/10.5281/zenodo.15382611, accessed on 11 May 2025.

## Abbreviations

BPPV	Benign Paroxysmal Positional Vertigo
ENT	Ear, Nose, and Throat
GP	General Practitioner
CME	Continuing Medical Education
CRP	Canalith Repositioning Procedure
